# Hemoglobin Genotype Frequencies in Lagos: Insights From Offspring of Hemoglobin AS Mothers

**DOI:** 10.7759/cureus.96325

**Published:** 2025-11-07

**Authors:** Adebukola K Orolu, Babawale T Bello, Adedoyin O Dosunmu, Ogunlade O Tolulope

**Affiliations:** 1 Department of Hematology, Alimosho General Hospital, Igando, Lagos, NGA; 2 Department of Hematology and Blood Transfusion, Eko University Of Medicine And Health Sciences (EkoUNIMED) Ijanikin, Lagos, NGA; 3 Department of Medicine, College of Medicine, University of Lagos, Idi-Araba, Lagos, NGA; 4 Department of Hematology and Blood transfusion, Eko University Of Medicine And Health Sciences (EkoUNIMED) Ijanikin, Lagos, NGA

**Keywords:** allele frequencies, as mother, gene frequency, hardy-weinberg equilibrium (hwe), hb genotype

## Abstract

Introduction

Sickle cell disease (SCD) is a genetic disorder with a high disease burden in many parts of sub-Saharan Africa. The disease follows an autosomal recessive inheritance pattern, and its persistence within populations is influenced by various genetic, biological, and environmental factors. In regions where carrier status is common, understanding hemoglobin (Hb) genotype distributions among newborns, particularly those born to Hb AS mothers, is crucial for genetic counseling and public health interventions. This study examines Hb genotype distributions and allele frequencies in neonates of Hb AS mothers in Lagos, Nigeria, and compares the observed distributions with Hardy-Weinberg Equilibrium (HWE) expectations derived from population-level allele frequencies.

Methods

A descriptive cross-sectional study was conducted at Alimosho General Hospital in Lagos, Nigeria. Blood samples were collected from 404 neonates born to Hb AS mothers and analyzed for Hb genotypes using the HemoTypeSC point-of-care test, while blood samples from mothers were assessed using cellulose acetate Hb electrophoresis and sickling test. Allele and genotype frequencies were calculated from observed distributions and compared with expected values under HWE. Expected genotype frequencies were derived using population-level allele frequencies as a reference model. Fisher’s exact and binomial tests (with 95% confidence intervals (CIs)) were applied to assess deviations between observed and expected values, with *p* < 0.05 considered statistically significant.

Results

Among 404 neonates born to Hb AS mothers, 190 (47.0%) had Hb AA, 188 (46.5%) had Hb AS, 20 (5.0%) had Hb SS, 5 (1.2%) had Hb AC, and 1 (0.2%) had Hb SC. Corresponding allele frequencies were Hb A: 0.709, Hb S: 0.284, and Hb C: 0.007. No significant deviation from the model-based expectations under HWE was observed (observed 5.0% vs expected 8.1%; p > 0.05). However, the combined frequency of high-risk genotypes (Hb SS and Hb SC) was 5.2% (95% CI: 3.2-7.9%), significantly lower than the 8.5% expected (p = 0.017).

Discussion

The observed Hb frequencies align closely with Hardy-Weinberg expectations, suggesting approximate equilibrium in this cohort. The slightly lower occurrence of high-risk genotypes (Hb SS and Hb SC) than predicted under the model (p = 0.017) may indicate subtle deviations from random mating, possibly influenced by partner selection preferences within the population. Although paternal genotypes were not determined, these findings provide useful insight into the potential value of strengthening premarital and antenatal genotype screening.

Conclusion

This study contributes to understanding Hb genotype inheritance among neonates of Hb AS mothers in Lagos. While genotype distributions largely followed Hardy-Weinberg expectations, findings point to possible social influences on mating patterns that warrant further exploration. Broader, couple-based, and population-level studies, incorporating paternal genotypes, are recommended to clarify the role of selective mating and demographic dynamics on genotype patterns in high-prevalence settings.

## Introduction

Sickle cell disease (SCD) is an inherited disorder associated with significant health challenges, particularly among individuals of African descent. In 2021, about 515,000 infants were born with SCD worldwide, with sub-Saharan Africa accounting for approximately 80% of these births [1]. In Nigeria alone, approximately 150,000 infants are born with SCD each year [2,3], accounting for nearly 30% of the total global cases and highlighting its considerable contribution to the global burden. The condition arises due to a mutation in the beta-globin gene, where valine replaces glutamic acid at the sixth amino acid position, resulting in the formation of an abnormal hemoglobin variant known as hemoglobin S (Hb S) [4]. In SCD, under conditions of low oxygen, Hb S tends to polymerize, causing the red blood cell to deform into a rigid and sticky, sickle-like shape. These altered cells have the tendency to obstruct blood flow in capillaries and venules, leading to various acute and chronic complications, including painful vaso-occlusive crises, hyperhemolytic anemia, aplastic anemia, and ischemic injuries to essential organs. These complications significantly reduce life expectancy [4]. The profound health challenges posed by SCD emphasize the necessity of advancing genetic research to enhance understanding of inheritance mechanisms and their implications for at-risk populations.

SCD is inherited in an autosomal recessive manner. When both parents carry the sickle cell trait (Hb AS), there is a 25% probability that their child will inherit two copies of the sickle cell gene (Hb SS), resulting in sickle cell anemia (SCA) [5]. Additionally, there is a 50% likelihood that the child will inherit one sickle cell gene and one normal gene (Hb AS), making them a carrier like their parents. The remaining 25% chance is that the child will inherit two normal hemoglobin genes (Hb AA), leading to no disease or carrier status [5]. In cases where a mother with Hb AS mates with an individual with Hb AA, the offspring has a 50% probability of inheriting the Hb AS genotype and a 50% probability of inheriting the Hb AA genotype, with no chance of developing Hb SS (0%) [5]. This pattern of inheritance follows the principles of the Hardy-Weinberg Equilibrium (HWE), expressed as $p^{2} + 2pq + q^{2} =1$, where p and q represent the frequencies of the normal (A) and sickle (S) alleles, respectively. The HWE model assumes random mating, no evolutionary pressures such as natural selection, and a sufficiently large population size. While these assumptions are useful for estimating expected genotype distributions in high-risk populations, actual population data may show minor deviations due to non-random mating or demographic factors influencing allele transmission [6,7].

Analyzing genotype frequencies is critical for both clinical practice and public health initiatives, particularly in Nigeria, where approximately 24% of the population is carriers of the sickle cell trait (Hb AS) [2]. In malaria-endemic regions, like Nigeria, the Hb AS genotype confers a survival advantage against malaria, which has contributed to the persistence of the Hb S gene in the population [8]. This high prevalence underscores the importance of understanding genotype distribution patterns in newborns to inform genetic counseling, prenatal screening programs, and public education campaigns. Several studies have explored the prevalence of carriers and SCD incidence across African populations [1,8]. Research indicates that in the Nigerian population, the genotype distribution of Hb AS and Hb SS is approximately 24% and 2%, respectively, whereas Hb AA ranges between 71% and 77% [2,9-12]. Other hemoglobin genotypes are less common. Despite these proportions being well-documented for the general population, data specifically describing Hb genotype frequencies among neonates of Hb AS mothers remain limited, particularly from hospital-based cohorts. This gap in data is significant given that Nigeria accounts for a large share of the global burden of SCD. Nigeria's unique genetic landscape, shaped by the interplay between the high carrier rate and the protective effect of the Hb AS genotype against malaria, raises questions about whether observed genotype frequencies in newborns align with predictions based on random mating models. Understanding these patterns is essential for designing effective genetic counseling and targeted educational programs as well as community-based screening efforts to support informed reproductive choices and reduce the incidence of SCD.

The objective of this study is to determine the distribution of hemoglobin genotypes and allele frequencies among neonates of Hb AS mothers in Alimosho General Hospital, Lagos State, Nigeria, and to compare the observed genotype frequencies with expectations derived from the HWE using population-level allele frequencies as reference. By comparing observed and expected genotype frequencies under random mating assumptions, this study is anticipated to provide valuable insights relevant to genetic counseling and SCD prevention efforts in Nigeria and other high-prevalence regions.

## Materials and methods

This research utilized a descriptive cross-sectional approach to evaluate the Hb genotype and allele frequencies among newborns delivered by AS mothers at Alimosho General Hospital, Lagos. As one of the 30 operational secondary healthcare facilities in Lagos State, this hospital is situated in the central area of Alimosho local government and offers a wide range of services, including maternal, child, and hematology care [13]. Prior to commencement of the study, ethical approval was obtained from the Human Ethics and Research Committee (HREC) of the Lagos University Teaching Hospital (LUTH), with ethical approval number: ADM/DCST/HREC/APP/4475.

Participant recruitment commenced in July 2021 and extended over an 18-month period. Inclusion criteria required mothers to have an Hb genotype of AS, be aged 18 years or older, and have delivered their babies at the study center while providing informed consent to participate. Exclusion criteria applied to postpartum mothers who declined to consent or whose newborns were over six weeks old at the time of recruitment. A total of 402 participants were included in the study, with the minimum sample size determined based on established guidelines for calculating sample sizes for populations with known proportions [14].

After delivery, postpartum mothers who met the eligibility criteria were approached for participation in the study while in the postpartum ward. Detailed explanations of the study’s purpose were provided in clear, accessible language, ensuring participants fully understood the research objectives. Written informed consent was then obtained, and any questions or uncertainties raised by the eligible mothers were addressed to their satisfaction. Recruitment continued consecutively until the required sample size was achieved.

Blood samples were collected from consenting mothers and used for Hb genotype analysis. The analysis was conducted using the cellulose acetate hemoglobin electrophoresis method as well as a sickling test. Hemoglobin electrophoresis was performed on cellulose acetate at an alkaline pH of 8.6 using Tris-EDTA-borate buffer. Electrophoresis runs were standardized with known Hb AA, AS, AC, SC, CC, and SS controls to ensure accurate band migration and interpretation. Results were cross-checked by two independent laboratory scientists, and doubtful results were repeated. The sickling test was conducted using freshly prepared sodium metabisulfite reagent, with positive and negative controls included in each run to confirm reagent performance. For the newborns of participating mothers, blood samples were obtained through a heel-prick procedure. These samples were immediately analyzed using the HemoTypeSC point-of-care diagnostic test, following the manufacturer’s guidelines [15]. Hemoglobin genotypes (AA, AS, AC, SS, and SC) were interpreted using a reference chart included with the test kit [15]. HemoTypeSC detects Hb A, Hb S, and Hb C with >99% sensitivity and specificity, even in the presence of high fetal hemoglobin (HbF) [15]. Although confirmatory methods such as isoelectric focusing (IEF) or high-performance liquid chromatography (HPLC) were not performed due to budget constraints, HemoTypeSC has been validated for neonatal screening and is considered reliable for detecting the major genotypes relevant to this study [15].

The data gathered during the study were analyzed using IBM SPSS Statistics for Windows, Version 28.0 (Armonk, NY: IBM Corp). Descriptive statistics, including frequencies and percentages, were used to effectively summarize the findings. Quantitative data were organized and presented in tabular format for clarity and ease of interpretation. Genotype frequencies were determined as the proportion of neonates with each genotype, while allele frequencies were derived from the observed genotype distributions using standard allele counting methods consistent with the Hardy-Weinberg principle. Expected genotype frequencies were calculated under HWE assumptions using population-level allele frequencies as a reference. Because the study cohort was restricted to neonates of Hb AS mothers, these expectations represent model-based approximations of random mating rather than exact Mendelian outcomes. To assess deviations between observed and expected genotype distributions, Fisher’s exact test was applied using a 5×2 contingency table comparing observed and expected counts for Hb AA, AS, AC, SS, and SC genotypes. Additionally, for the combined frequency of high-risk genotypes (Hb SS and Hb SC), an exact binomial confidence interval was computed, and a binomial test was performed to evaluate the difference from expected frequencies under the HWE model for HbAS mothers. This method is particularly suitable for datasets with multiple alleles and rare genotypes, as it provides more accurate and reliable results [16]. The level of statistical significance for all analyses was established at p < 0.05.

## Results

Table 1 shows the socio-demographic characteristics of mothers (n = 402). The mean maternal age was 31.0 ± 3.5 years, with ages ranging from 18 to 40 years. The majority of participants were of Yoruba ethnicity (64.9%), followed by Igbo (34.9%) and a small proportion of Hausa (0.2%).

**Table 1:** Socio-demographic characteristics of mothers

Variable (n = 402)	Frequency (Percentage)
Maternal age (years)	Mean ± SD: 31.0 ± 3.5; Range: 18–40
Ethnicity	
Yoruba	262 (64.9%)
Igbo	141 (34.9%)
Hausa	1 (0.2%)
Education status	
Primary	10 (2.5%)
Secondary	111 (27.6%)
Tertiary	281 (69.9%)
Employment status	
Employed	345 (85.8%)
Unemployed	57 (14.2%)
Marital status	
Married	392 (97.5%)
Single	10 (2.5%)

Regarding educational attainment, 69.9% of participants had a tertiary education, 27.6% had completed secondary education, while only 2.5% had a primary education (Table 1). Employment status revealed that most participants (85.8%) were employed, and 14.2% were unemployed. Nearly all participants were married (97.5%), with only 2.5% being single (Table 1).

As shown in Table 2, a majority of participants (82.9%) reported being aware of their partner’s hemoglobin genotype, while 17.1% were not aware.

**Table 2:** Awareness of partner’s hemoglobin genotype among mothers (n = 402)

Partner Genotype Awareness	Frequency (Percentage)
Yes	335 (82.9%)
No	69 (17.1%)

Even though a total of 402 mothers with the Hb AS genotype participated in the study, 2 mothers gave birth to twins, resulting in a total of 404 newborns. Among these newborns, 190 (47.0%) were found to have the Hb AA genotype, 5 (1.2%) were identified as Hb AC, and 188 (46.5%) were Hb AS (Table 3). Additionally, Hb SS was demonstrated in 20 neonates (5.0%), and one neonate (0.2%) had Hb SC (Table 3).

**Table 3:** The Hb genotype in the cohort of babies born to AS mothers

Hb Genotype	Number of Neonates (n = 404)	Percentage (%)
AA	190	47.0
AC	5	1.2
AS	188	46.5
SC	1	0.2
SS	20	5.0

The distribution of hemoglobin genotypes among the neonates indicates that homozygosity for Hb AA was observed in 47% of the cases. Heterozygous genotypes, including Hb AC and Hb AS, collectively accounted for 47.7%, while the proportions of high-risk genotypes associated with SCD, specifically Hb SS and Hb SC, were found in 5.2% of the newborns.

The frequencies of hemoglobin genotypes among the neonates are presented in Table 4. The most common genotypes were Hb AA (0.470) and Hb AS (0.465), together accounting for the majority of the cohort. The Hb SS genotype, which is associated with SCA, had a frequency of 0.050. Less common genotypes include Hb AC (0.012) and Hb SC (0.002).

**Table 4:** Frequency (f) of hemoglobin genotypes among neonates born to AS mothers

Hb Genotype	Number of Neonates (n = 404)	Frequency (f)
AA	190	0.470
AC	5	0.012
AS	188	0.465
SC	1	0.002
SS	20	0.050

The genotype frequency (f) is calculated using the relative frequency formula:

\[\mathcal{f}(\text{genotype}) = \frac{\text{number of individuals with a specific genotype}}{\text{total number of individuals in the study population}}\]

The allele frequencies (f) of Hb A, Hb S, and Hb C in the cohort of babies born to AS mothers were thereafter derived from the genotype frequencies using the allele frequency calculation formula under the Hardy-Weinberg principle [6]:

\[f(\text{allele}) = f(\text{homozygous genotype}) + \frac{1}{2} f(\text{heterozygous genotype})\]

Frequency of allele A

\[f(A) = f(\text{Hb AA}) + \frac{1}{2} \left[ f(\text{Hb AC}) + f(\text{Hb AS}) \right]\] \[f(A) = 0.470 + \frac{1}{2} (0.012 + 0.465)\] \[f(A) = 0.470 + \frac{0.477}{2}\] \[f(A) = 0.470 + 0.2385 = 0.709\]

Frequency of allele S

\[f(S) = f(\text{Hb SS}) + \frac{1}{2} \left[ f(\text{Hb AS}) + f(\text{Hb SC}) \right]\] \[f(S) = 0.050 + \frac{1}{2} (0.465 + 0.002)\] \[f(S) = 0.050 + \frac{0.467}{2}\] \[f(S) = 0.050 + 0.2335 = 0.284\]

Frequency of allele C

\[f(C) = f(\text{Hb CC}) + \frac{1}{2} \left[ f(\text{Hb AC}) + f(\text{Hb SC}) \right]\] \[f(C) = 0 + \frac{1}{2} (0.012 + 0.002)\] \[f(C) = \frac{0.014}{2}\] \[f(C) = 0.007\]

The allele frequencies of Hb A, Hb S, and Hb C among neonates born to Hb AS mothers are presented in Table 5. The Hb A allele was the most frequent, with a frequency of 0.709, followed by the Hb S allele at 0.284, while the Hb C allele was the least common, with a frequency of 0.007. 

**Table 5:** Allele frequencies of HbA, HbS, and HbC among neonates born to AS mothers

Variable	Allele frequency (f)
Allele A	0.709
Allele S	0.284
Allele C	0.007

The allele frequencies of A, S, and C are, respectively:

\[f(A) = p, \quad f(S) = q \quad and \quad f(C) = r\] 

Total genotype frequency is: \[f(\text{Hb AA}) + f(\text{Hb SS}) + f(\text{Hb CC}) + f(\text{Hb AS}) + f(\text{Hb AC}) + f(\text{Hb SC}) = p^2 + q^2 + r^2 + 2pq + 2pr + 2qr = 1\]

Using the allele frequencies calculated from the study cohort, expected Hb genotype frequencies were estimated under HWE assumptions to provide a model-based reference for comparison with the observed distributions and calculated as:

Expected Frequency of Hb AA:

\[f(\text{Hb AA}) = f(A)^2 = (0.709)^2 = 0.503\]

Expected Frequency of Hb AS:

\[f(\text{Hb AS}) = 2 \times f(A) \times f(S) = 2 \times 0.709 \times 0.284 = 0.403\]

Expected Frequency of Hb SS:

\[f(\text{Hb SS}) = f(S)^2 = (0.284)^2 = 0.081\]

Expected Frequency of Hb AC:

\[f(\text{Hb AC}) = 2 \times f(A) \times f(C) = 2 \times 0.709 \times 0.007 = 0.010\]

Expected Frequency of Hb SC:

\[f(\text{Hb SC}) = 2 \times f(S) \times f(C) = 2 \times 0.284 \times 0.007 = 0.004\]

Expected Frequency of Hb CC:

\[f(\text{Hb CC}) = f(C)^2 = (0.007)^2 = 0.00005\]

Table 6 presents the expected hemoglobin genotype frequencies for neonates of Hb AS mothers under HWE assumptions, incorporating population-level paternal allele frequencies to provide a model-based reference for comparison with observed data. The Hb AA genotype was expected to have the highest frequency at 0.503 (50.3%), followed by Hb AS at 0.403 (40.3%). The expected frequency of Hb SS was 0.081 (8.1%). The rarer genotypes, Hb AC and Hb SC, were predicted at 0.010 (1.0%) and 0.004 (0.4%), respectively, while the Hb CC genotype was nearly absent (0.00005 or 0.0%).

**Table 6:** Expected Hb genotype frequencies in the newborns born to AS mothers under HWE assumptions (n = 404) HWE: Hardy-Weinberg Equilibrium

Hb Genotype	Hb Genotype Frequency (f)	N (Percentage)
AA	0.503	203 (50.3)
AC	0.010	4 (1.0)
AS	0.403	163 (40.3)
SC	0.004	1 (0.4)
SS	0.081	33 (8.1)
CC	0.00005	0 (0.0)

The expected proportions of high-risk genotypes associated with SCD (Hb SS and Hb SC) in the cohort are 0.085 (8.5%). A binomial test indicates a statistically significant difference (p = 0.017) between the observed frequency of high-risk genotypes (Hb SS and Hb SC) (5.2%; 95% CI: 3.2-7.9%) and the expected frequency (8.5%) under HWE.

The analysis of the expected and observed outcomes in neonates using Fisher's exact test yielded a p-value of 0.2388 (Table 7), indicating no statistically significant difference between the expected and observed genotype frequencies (p > 0.05).

**Table 7:** Comparison of the expected HWE Hb genotype proportions and that of the study data HWE: Hardy-Weinberg Equilibrium *p-value was derived from Fisher’s exact test p<0.05 is statistically significant

Hb Genotype	Observed Proportions in Study (n = 404)	Expected HWE Proportions (n =404)	p-value
AA	190	203	0.2388*
AC	5	4	
AS	188	163	
SC	1	1	
SS	20	33	

## Discussion

The study involved 402 Hb AS mothers and examined the hemoglobin genotype distribution among 404 neonates. The dominant genotypes, Hb AA and Hb AS, together comprised 93.5% of the cohort of neonates (190 Hb AA and 188 Hb AS), which is consistent with data in the Lagos region, where these genotypes account for over 95% of the population [2,9-12].

Allele frequency calculations revealed values of A - 0.709, S - 0.283, and C - 0.007, demonstrating that the Hb C variant is rare in Nigeria; a pattern consistent with previous studies [17,18]. Nevertheless, the detection of Hb AC and Hb SC genotypes, even at low frequencies, reflects the presence of genetic diversity at the hemoglobin locus. Given the low prevalence of the Hb C allele and the more prevalent S allele in this study, the lower frequency of Hb AC compared to Hb AS may be attributed to reduced co-inheritance of A and C alleles relative to A and S alleles [12]. Understandably, no cases of Hb CC were identified.

The HWE principle assumes that allele and genotype frequencies in a population remain stable across generations in the absence of evolutionary forces such as natural selection, emergence of mutations, genetic drift, gene flow, consanguinity, or non-random mating. The most important of these are consanguinity and gene flow [19]. The study data showed that the expected HWE Hb genotype frequencies of Hb AA, AS, SS, AC, and SC are 0.503 (50.3%), 0.403 (40.3%), 0.081 (8.1%), 0.010 (1.0%), and 0.004 (0.4%), respectively. No Hb CC genotypes were expected, consistent with the low Hb C allele frequency in the cohort and in the environment [17,18,20]. Statistical comparison of observed and expected genotype frequencies showed no significant deviations (p > 0.05), indicating that the distribution of Hb genotypes among neonates of Hb AS mothers was consistent with the expected pattern under the model used. This finding is interpreted within the context of the study design. Furthermore, because the cohort was restricted to neonates of Hb AS mothers at a single center, the analysis reflects model-based expectations rather than population-wide equilibrium. The absence of deviation may therefore indicate approximate consistency with random mating assumptions in this group, but not necessarily genetic equilibrium across the broader population. In contrast, a systematic review in Nigeria reported significantly higher-than-expected Hb SS cases [19], attributed to consanguinity, particularly in the northern Hausa region. However, this pattern was not observed in the southwestern Yoruba region where the present study was conducted [19,21].

Although fathers were not genotyped in the study, a high proportion of mothers (86%) reported awareness of their partner’s Hb genotype, and most had attained at least secondary (69.9%) or tertiary (27.6%) education, suggesting a relatively high level of health literacy. While such awareness alone does not confirm non-random mating, it may reflect the broader impact of genetic counseling and public education on partner selection practices. Awareness and education may encourage informed partner choice, with individuals intentionally avoiding partners who are also carriers of the sickle cell allele, thereby reducing the likelihood of Hb SS inheritance in offspring [22]. Additionally, the observed A to S allele frequency ratio of 2.5:1 may further suggest possible non-random inheritance patterns. Previous studies in Nigeria have documented a preference for Hb AA partners among Hb AS individuals [23,24], contributing to lower Hb SS frequencies [20,23]. Similar trends have also been reported across sub-Saharan Africa, shaped by genetic awareness, healthcare access, and sociocultural attitudes toward individuals with SCD [1,22].

This study documents that the combined observed frequency of high-risk genotypes (Hb SS and Hb SC) was 5.2% (21/404; 95% CI 3.2-7.9%), compared with 8.5% computed under the HWE model for AS mothers. A binomial test showed that this difference was statistically significant (p = 0.017). Despite paternal genotypes not being assessed, the result may nonetheless reflect subtle deviations from random mating or other demographic influences on allele transmission.

For Hb AS, the observed frequency is 0.465, which is slightly higher than the expected frequency of 0.403. Although this difference was not statistically significant, these findings support the relevance of genetic counseling and genotype-based mate selection in influencing Hb genotype distribution. While the World Health Organization (WHO) recommends newborn screening for SCD, particularly in low-resource settings where access to specialized care is limited, such programs are not yet widely implemented in Nigeria. This limits their effectiveness for large-scale prevention [20]. Similarly, prenatal diagnostic techniques or curative interventions such as gene therapy are inaccessible due to high costs and limited availability. In contrast, premarital genetic counseling currently remains the most accessible, cost-effective, and most practical strategy for long-term SCD prevention in sub-Saharan Africa. It can be implemented at the community level without requiring advanced healthcare infrastructure. By educating couples on their Hb genotype compatibility before marriage or parenthood, this approach empowers individuals with knowledge of their genetic risk, reducing transmission of SCD to offspring [20].

Despite this study offering valuable insights into Hb genotype distribution and Hardy-Weinberg dynamics, a few limitations are noted. The low frequency of certain genotypes (Hb AC, Hb SC) and the absence of Hb CC reduced the statistical power to analyze these variants. Its single-center, cross-sectional design limits generalizability and prevents assessment of temporal trends. Furthermore, paternal genotypes were not directly assessed, and this limits the precision of deviation testing from model expectations.

## Conclusions

This study provides new insights into the distribution of Hb genotypes among neonates of Hb AS mothers in Lagos, highlighting patterns that align closely with Hardy-Weinberg expectations based on population-level allele frequencies. While findings suggest no strong deviations from random mating within this cohort, the restricted design limits broader inference. Nonetheless, these data establish a basis for further couple-based studies that incorporate paternal genotypes, multi-regional samples, and longitudinal designs to better understand genotype inheritance patterns and their public health implications in high-prevalence settings.
